# The Association between Online Learning and Food Consumption and Lifestyle Behaviors and Quality of Life in Terms of Mental Health of Undergraduate Students during COVID-19 Restrictions

**DOI:** 10.3390/nu14040890

**Published:** 2022-02-20

**Authors:** Charoonsri Chusak, Mutthatinee Tangmongkhonsuk, Jutaporn Sudjapokinon, Sirichai Adisakwattana

**Affiliations:** Phytochemical and Functional Food Research Unit for Clinical Nutrition, Department of Nutrition and Dietetics, Faculty of Allied Health Science, Chulalongkorn University, Bangkok 10330, Thailand; charoonsri.c@gmail.com (C.C.); mutthatinee.t@gmail.com (M.T.); jutaporn.sudjapokinon@gmail.com (J.S.)

**Keywords:** online learning, food consumption behavior, lifestyle, quality of life, undergraduate students, COVID-19 restrictions

## Abstract

The COVID-19 pandemic caused the abrupt replacement of traditional face-to-face classes into online classes. Several studies showed that online teaching and learning produced adverse mental health for students. However, no research has been conducted so far analyzing the association between the duration of online and food consumption and lifestyle behaviors and quality of life in terms of mental health of undergraduate students. This study aimed to determine the association between the duration of online learning and food consumption behaviors, lifestyles, and quality of life in terms of mental health among Thai undergraduate students during COVID-19 restrictions. A cross-sectional online survey of 464 undergraduate students was conducted at Chulalongkorn University, Bangkok, Thailand, between March and May 2021. The majority of undergraduate students stated that they spent 3–6 h per day on online learning (76.1%) and used their digital devices such as computers, tablets, or smartphones more than 6 h per day (76.9%). In addition, they had 75.4% of skipping breakfast (≥3 times/week) and 63.8% of sleep duration (6–8 h/day). A higher proportion of students who drank tea or coffee with milk and sugar while online learning was observed. The results found that the increased duration of online learning was significantly associated with skipping breakfast and the frequency of sugary beverage consumption. On the other hand, the increased computer, tablet, and smartphone usage for online learning was correlated with lower sleep duration and a poor quality of life in terms of mental health. The findings from this study contribute to a report of the association between online learning and food consumption and lifestyle behaviors and quality of life of undergraduate students, emphasizing the necessity for intervention strategies to promote healthy behaviors.

## 1. Introduction

The World Health Organization (WHO) declared the outbreak of a new Coronavirus disease (COVID-19), severe acute respiratory syndrome coronavirus 2 (SARS-CoV-2), as a pandemic on 11 March 2020 [1]. With the ongoing COVID-19 pandemic, the number of daily confirmed COVID-19 cases has increased dramatically in many regions, leading to unprecedented disruption in economic and healthcare systems all over the world [2]. Evidence reveals that the COVID-19 pandemic induces an impairment in quality of life in terms of mental health for the general population [3,4,5,6,7]. As part of the worldwide pandemic, Thailand has witnessed three waves of COVID-19 outbreaks between 2020 and 2021 [8,9]. As a result of the crisis, the universities temporarily closed their offices and canceled all physical classes, transitioning from face-to-face sessions to an online teaching and learning mode. 

Several reports have shown that online teaching and learning during COVID-19 pandemic circumstances creates mental health problems and negative effects on quality of life of students worldwide [10,11,12,13]. For example, low quality of life and a higher frequency of depression and/or anxiety were observed in university students while online learning [14]. During the COVID-19 restriction period, they are forced to stay in a closed environment to attend classes for several hours each day and lack direct social connections [15]. Online learning platforms have also increased the duration of computer, tablet, and smartphone usage, the study load and volume of tasks and assignments, leading to lack of sleep, and destructive eating behaviors [15,16]. Moreover, online learning during the COVID-19 pandemic resulted in changes in food intake and lifestyle habits, including increased consumption of unhealthy diets such as sugary beverages, snacks, and sweets and decreased physical activity and exercise [17,18,19]. Furthermore, it has been observed that online learning university students consumed snacks and skipped breakfast during the pandemic [18]. Although several reports on online learning education linked to eating habits, lifestyles, and mental health during the COVID-19 pandemic have been published, studies regarding the association between the duration of online learning and students’ food consumption and lifestyle habits and quality of life during COVID-19 restrictions are not yet understood. Consequently, we hypothesized that the increased duration of online learning and computer, tablet, and smartphone usage might be related to undergraduate students’ eating and lifestyle behaviors and quality of life related to mental health. Therefore, the objective of the current study was to determine the association between the duration of online learning and computer and mobile learning device usage and the frequency of food consumption, lifestyle habits, and quality of life in terms of mental health among Thai undergraduate students under COVID-19 restrictions.

## 2. Materials and Methods

### 2.1. Study Design and Participants

This research was a cross-sectional descriptive study conducted during the COVID-19 restriction period in Thailand. Undergraduate students (age > 18 years) who studied online learning classes at Chulalongkorn University, Bangkok, Thailand, between March and May 2021, were recruited through social media such as Facebook, Line, and Instagram. An online survey operated through Google document form in the Thai language, and the participant information sheet and consent form were distributed to the participants by e-mail, Facebook, and Line. Participants were excluded from the data analysis if they did not answer the questionnaire completely. The study protocol was performed following the Declaration of Helsinki, approved by the Research Ethics Review Committee for Research Involving Human Research Participants, Chulalongkorn University (COA No. 064/2564).

### 2.2. Questionnaire for Surveys

The questionnaire was divided into four sections: sociodemographic characteristics, food consumption behaviors, lifestyles, and quality of life. The survey contained 36 questions composed of multiple choices, blanks, and a rating scale on the impact of online learning. The web-based questionnaire was sent to a group of five experts in nutrition and public health to evaluate the validity and reliability of each question included. The Cronbach’s alpha index of the overall instrument had reliability (alpha = 0.7).

The anonymous questionnaire included sociodemographic characteristics, anthropometrics, income, accommodation, and study area. The duration of online learning is correlated to the duration of computer, tablet, and smartphone usage for online learning. Therefore, participants were asked to self-record the number of hours that they spent online studying and using computers and mobile learning devices for the study. Thereafter, the duration of online learning (3 h/day, 3–6 h/day, or >6 h/day) and computer, tablet, and smartphone usage (6 h/day, 6–9 h/day, or >9 h/day) for online learning were classified in the categorical group based on the credit registration criteria for undergraduate students at Chulalongkorn University. 

In the lifestyle behavior assessment, respondents were required to allude the duration of exercise (no exercise, <3 times or 150 min/week or ≥3 times or 150 min/week) following the WHO guideline [20], the duration of sleep (<6 h/night, 6–8 h/night or >8 h/night) according to the previous report [21], self-cooking, and skipping breakfast (≥3 times/week, <3 times/week or none). The specific lifestyle behaviors assessed were eating food, snacks, or beverages while online learning. In addition, participants were asked to provide the types of snacks and beverages that they ate during the online study. 

The food consumption questionnaire was developed from the Thai Food-Based Dietary Guideline, Department of Health, Ministry of Public Health, Thailand, to reflect local food consumption patterns [22]. Participants were asked to self-recall their frequency of food consumption in the previous two weeks during learning online under COVID-19 restrictions. The food consumption of fruits and vegetables, high-fat diets, snacks, western diets, sugary beverages, and instant foods were asked to be chosen from one of the four categories of frequency (none, 1–2 days/week, 3–4 days/week, 5–6 days/week or every day). Data were dichotomized as infrequently (≤4 days/week) and frequently (>4 days/week). During the same period, they were also asked to report fresh vegetable consumption (≤4 servings/day or more), and fruit consumption (≤3 servings/day or more).

The quality of life score in terms of mental health was assessed using the World Health Organization Quality of Life (WHOQOL), comprising six questions on the individual’s perceptions related to mental health. A Likert scale of 5-points scored all questions asked about the overall quality of life related to mental health. The total score of quality of life ranges from 6 to 14 (poor), 15–22 (fair), and 23–30 (good).

### 2.3. Sample Size Calculation

The sample size estimation was performed according to previously described approaches [23]. The sample size was calculated based on a total undergraduate student population of Chulalongkorn University in 2020 with a confidence interval of 95% and a margin of error of 5%. The minimum sample size required was 379 undergraduate students. Taking a 20% nonresponse rate into consideration, the total sample size was calculated to be 455.

### 2.4. Statistical Analysis

Data were represented as numbers and percentages in parentheses for categorical variables (%). A chi-square test (χ2) was used to determine the relationship between categorical variables, with *p* < 0.05 being statistically significant. Multinomial logistic regression was used to predict the association between dependent variables (duration of online learning and duration of computer, tablet, and smartphone usage for online learning) and more independent variables (frequency of sugary beverage consumption, skipping breakfast, duration of sleep, and quality of life in mental health).

## 3. Results

### 3.1. Sociodemographic Variables and Food Consumption and Lifesyle Behaviors

As shown in Table 1, a total of 480 undergraduate students representing all areas of the study responded to the survey. After validation of the data, 464 respondents were included in the study, with 69.2% and 38.2% of females and males, respectively. Sixteen participants who did not answer the questionnaire completely were excluded from the study. Among the participants, 47.2% had a body mass index (BMI) between 18.5 and 22.9. Most undergraduate participants’ responses to the questionnaire were in the health sciences area of the study (45.7%) and living with their parents (56.3%).

**Figure 1:**
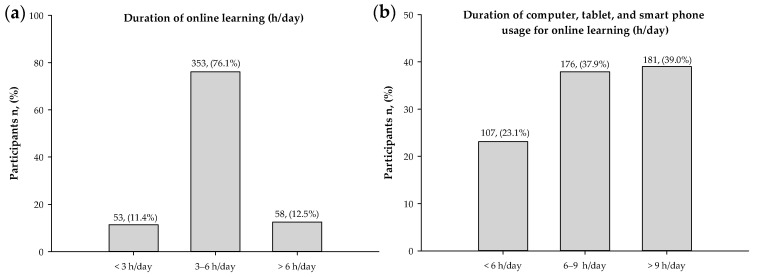
Percentages of respondents for (**a**) duration of online learning and (**b**) duration of computer, tablet, and smartphone usage for online learning (n = 464).

As demonstrated in Figure 1, most undergraduate students stated their duration of online learning at 3–6 h/day (76.1%) and the duration of computer, tablet, and smartphone usage for online learning at 6–9 h/day (37.9%) and >9 h/day (39%).

With regards to lifestyle, most of the population declared they were non-smokers (98.9%) while they had 75.4% of skipping breakfast (≥3 times/week), the duration of sleep (6–8 h/day) (63.8%), and no self-cooking (60.6%), as shown in Table 2. Furthermore, 53.7% of students did not exercise, while only 14.4% described some exercise (3 times per week or 150 min per week).

Table 3 describes the frequency of food consumption among undergraduate students. In terms of eating habits, only 29.1 % of undergraduate students consumed fruit and vegetables daily (>4 days/week), while 70.9% consumed them less than four days/week. Furthermore, most participants (67.7%) consumed fresh vegetables less than four servings/day, whereas 59.3% of students ate fruits less than three servings/day. Interestingly, participants stated the frequency of unhealthy food consumption (≤4 days/week), including a high-fat diet (77.2%), a western diet (95.0%), sugary beverages (68.5%), and instant food (95.5%). 

Table 4 demonstrates eating behaviors and types of snacks and beverages consumed by respondents during online learning. A total of 65.5% of participants declared that they did not consume food or snacks while learning online. Among those who consumed snacks, they ate prepared foods (9.9%), ready-to-eat savories (33.59%), bakery wares (20.83%), confectionery (19.27%), and fruits, vegetables, seaweed, nuts, and seeds (9.38%).

Surprisingly, a higher proportion of participants who drank beverages during online learning (81.5%) was reported. Most of the participants (30.52%) drank tea or coffee with milk and sugar, milk (16.56%), soft drinks (12.12%), juices (11.81%), milk tea (8.9%), sugar-free tea or coffee (4.91%), and others (6.6%).

### 3.2. The Association between Online Learning and Food Consumption and Lifestyle Behaviors and Quality of Life in Terms of Mental Health

Supplementary Table S1 demonstrates the distribution of the food consumption behaviors, lifestyles, and quality of life in the mental health of participants by the duration of online learning. There was an association between the duration of online learning and food consumption behaviors (frequency of sugary beverage consumption and skipping breakfast). The percent frequency of sugary beverages (>4 days per week) was significantly higher in participants who studied online for less than 3 h/day. Nevertheless, when the duration of online learning was increased, this percent distribution (≤4 days/week) was considerably lower.

As shown in Table 5, the odds ratio for the duration of online learning (3–6 h/day) was 0.42 times lower among those who had a frequency of sugary beverage consumption (>4 days/week) when compared to the duration of online learning (<3 h/day).

In addition, a lower proportion of participants who skipped breakfast (≥3 times/week) was also found when increasing the duration of online learning by more than 3 h/day. Furthermore, the odds ratio for skipping breakfast was 0.29 times lower among participants with a longer duration of online learning (>6 h/day) when compared to the group that studied online learning for less than 3 h/day.

Supplementary Table S2 shows the distribution of the food consumption behaviors, lifestyles, and quality of life in the mental health of participants by the duration of computer, tablet, and smartphone usage for online learning. Our analysis also demonstrated an association between the duration of computer, tablet, and smartphone usage for online learning and the duration of sleep. The number of participants with a long duration of sleep (>6 h/day) was shown to be low in the groups who spent their time on computers, tablets, and smartphones for learning more than 6 h/day. 

When compared to the groups using digital devices (<6 h/day), the odds ratio for the duration of sleep (<6 h/day) was 3.52 and 4.62 times higher among undergraduate students who used a computer, tablet, or smartphone for online learning for 6–9 h/day and more than 9 h/day, respectively (Table 6).

There was an association between the duration of computer, tablet, and smartphone usage for online learning and quality of life in terms of mental health (Supplementary Table S2). When increasing the duration of computer, tablet, and smartphone usage, a lower proportion of students demonstrating fair and good quality of life occurred, while a higher proportion of students manifested a poor quality of life. 

As shown in Table 6, 3.74 and 5.29 times the odds were identified as poor quality of life in the participants who had a duration of computer, tablet, and smartphone usage for online learning of 6–9 h/day and more than 9 h/day, respectively, when compared to the students who used digital devices for less than 6 h/day.

## 4. Discussion

This study is the first cross-sectional, web-based study investigating the relationships between online learning and food consumption and lifestyle behaviors and quality of life in terms of mental health of undergraduate students during the implementation of the COVID-19 restriction without affecting the country’s lockdown policy. According to the lifestyle behavior assessment, 75.4% of undergraduate students had breakfast skipping behavior more than three times per week. Argun et al. stated that adults skip breakfast more frequently than other main meals because of lack of time, waking up late, and fatigue [24]. These results are similar to previous studies demonstrating that more than half of the surveyed respondents had meal skipping behavior as undergraduate students who studied online during the COVID-19 pandemic [18]. Under the lockdown, the reason behind the meal skipping behavior of students may be attributed to the low accessibility of food purchased from physical stores [25]. 

Interestingly, we found an association between the duration of online learning and breakfast skipping behavior during COVID-19 restrictions without the lockdown policy. When the duration of online learning was increased, the proportion of participants who skipped breakfast (≥3 times/week) was considerably lower. The proportion of undergraduate students who engaged in extensive online learning (>6 h/day) skipped breakfast (≥3 times/week) less than other participants who studied online (<3 h/day). It may be because undergraduate students must continuously participate in the online class throughout the day. Consequently, they consume breakfast, which may help to reduce excessive hunger and appetite while learning online. In addition, the COVID-19 crisis has forced university closures, and students are not required to access campus for classes and other learning activities, leading to having more time for breakfast consumption at home.

Our study also revealed an association between the duration of online learning and the frequency of sugary beverage consumption. When students increased their online learning time (3–6 h per day) compared to the control group (3 h per day), their consumption of sugary beverages decreased (>4 days per week). The actual reasons for supporting the discovery remain unknown. However, most students reported drinking tea or coffee with milk and sugar while learning online, which was a surprising finding. Today, milky tea and coffee with sugar have gained more popularity in Southeast Asia. Recent studies revealed that this beverage contained a high sugar composition (sucrose, fructose, and glucose) recognized as sugar-sweetened beverages (SSB) [26,27]. Considering the components of this beverage, adding ingredients like jelly, boba, and egg pudding to SSB can result in an increase in total calories per serving size from 299 to 515. According to this viewpoint, excessive SSB consumption may contribute to increased risk factors precipitated by adverse glycemic effects, as well as higher rates of overweight, obesity, and cardiometabolic diseases in online learning undergraduate students [28,29].

Today, the COVID-19 pandemic has a negative impact on the general population’s quality of life, particularly in terms of physical, social, psychological, mental, and spiritual health [30,31,32]. In this study, six significant themes of the World Health Organization Quality of Life (WHOQOL) associated with mental health problems were chosen, indicating an individual’s perception of well-being in the context of satisfaction with essential aspects of life. Current findings reveal that spending more time online learning through digital device usage was significantly correlated with a rise in poor quality of life in terms of mental health and sleep deprivation among undergraduate students. Current findings reveal that spending more time online learning through computer, smartphone, and tablet usage was significantly correlated with a rise in poor quality of life in terms of mental health and sleep deprivation among undergraduate students. These results are consistent with other studies regarding online learning and mental health under the COVID-19 restriction [33,34]. For example, students had a higher risk of poor mental health and stress due to their shift from face-to-face learning to an online learning system during the COVID-19 pandemic [33,34,35,36]. Furthermore, online study contributes to mental health issues including increased anxiety and absenteeism among students [33,34]. In addition, worsened health-related behaviors such as a reduction in physical activities and daily consumption of fruits and vegetables, together with increased hours of computer and smartphone usage, have also been observed in students during the COVID-19 confinement [37,38,39]. The online learning activities may comprise receiving instruction and many assignments, presentations, reports, and exams, leading to the stressful load of work required. These activities force students to stay up late completing assignments using computers, smartphones, and tablets, resulting in shorter sleep duration and poor quality of life in terms of mental health. It is important to point out that the quality of life score in terms of mental health could not indicate the level of stress among online undergraduate students. Further studies are needed to investigate the effects of the duration of online learning on the level of perceived stress in undergraduate students.

This study has several limitations to acknowledge. First, using an online self-administered questionnaire, we could not conclude any significant causality between any variables studied. Second, there are no questions in the survey concerning undergraduate students’ food consumption behaviors, lifestyles, and quality of life before implementing COVID-19 restrictions. Due to a lack of comparative data, the present study could not evaluate undergraduate students’ changing lifestyle and food consumption behaviors caused by the COVID-19 restriction. Third, our targeted participants were undergraduate students who studied online learning at a bachelor’s level in Bangkok, the capital city of Thailand. The findings of this study may not be generalizable to undergraduate students in urban and rural areas of Thailand because of differences in culture and eating behaviors.

The current findings have several implications for research and practice. For instance, we suggest that specific and nutritional education programs, especially dietary guidelines and awareness programs, could be initiated to advise undergraduate students who drink unhealthy beverages and skip breakfast. In line with this, the programs must emphasize the importance of regular meal consumption and healthier snack and beverage selections to promote changes in food consumption and lifestyle behaviors among online learning undergraduates. Moreover, university policymakers need to explore and establish the procedures and mechanisms for responding to these findings with immediate mental health assistance for at-risk students. For example, online learning programs should limit screening times to reduce sedentary behavior. Meanwhile, offering other health education programs for undergraduate students highlights increasing physical activity and a decreasing level of stress. 

## 5. Conclusions

According to the findings, the increased duration of online learning was associated with a decreased frequency of breakfast skipping and sugary beverage consumption. Meanwhile, the increased duration of computer, tablet, and smartphone usage for online learning was also correlated with a decreasing duration of sleep and a poor quality of life in mental health. Thus, understanding factors associated with the duration of online learning and the duration of digital device usage may provide information that will be useful in developing health promotion programs aimed at changes in food consumption and lifestyle behaviors and improving quality of life during the COVID-19 restrictions.

## Figures and Tables

**Table 1:** Socio-demographics of undergraduate students (n = 464).

Variables	Participants, n (%)
Gender	
Female	321 (69.2)
Male	143 (30.8)
BMI (kg/m^2^)	
<18.5	107 (23.1)
18.5–22.9	219 (47.2)
≥23.0	138 (29.7)
Age (years)	
18	25 (5.4)
19	66 (14.2)
20	98 (21.1)
21	134 (28.9)
22	99 (21.3)
≥23	42 (9.1)
Income (Baht/month)	
<5000	149 (32.1)
5001–10,000	217 (46.8)
>10,000	98 (21.1)
Living	
With parents	261 (56.3)
With friend	111 (23.9)
Alone	91 (19.8)
Area of study	
Health Sciences	212 (45.7)
Sciences and Technology	136 (29.3)
Social Sciences and Humanities	116 (25.0)

Notes. n = numbers of participant; BMI = body mass index.

**Table 2:** Lifestyle behaviors of undergraduate students (n = 464).

Variables	Participants, n (%)
Duration of exercise	
No exercise	249 (53.7)
<3 times or 150 min/week	148 (31.9)
≥3 times or 150 min/week	67 (14.4)
Duration of sleep (h/night)	
<6	144 (31.0)
6–8	296 (63.8)
>8	24 (5.2)
Smoking	
No	459 (98.9)
Yes	5 (1.1)
Self-cooking	
No	281 (60.6)
Yes	183 (39.4)
Skipping breakfast	
<3 times/week or none	114 (24.6)
≥3 times/week	350 (75.4)

Notes. n = numbers of participant.

**Table 3:** The frequency of food consumption of undergraduate students (n = 464).

Variables	Participants, n (%)
Frequency of fruits and vegetables	
≤4 days/week	329 (70.9)
>4 days/week	135 (29.1)
Fresh vegetable consumption	
<4 servings/day	314 (67.7)
≥4 servings/day	150 (32.3)
Fruit consumption	
<3 servings/day	275 (59.3)
≥3 servings/day	189 (40.7)
Frequency of high-fat diet consumption	
≤4 days/week	358 (77.2)
>4 days/week	106 (22.8)
Frequency of snack consumption	
≤4 days/week	404 (87.1)
>4 days/week	60 (12.9)
Frequency of western diet consumption	
≤4 days/week	441 (95.0)
>4 days/week	23 (5.0)
Frequency of sugary beverage consumption	
≤4 days/week	318 (68.5)
>4 days/week	146 (31.5)
Frequency of instant food consumption	
≤4 days/week	445 (95.9)
>4 days/week	19 (4.1)

Notes. n = numbers of participant.

**Table 4:** Eating behaviors and types of snacks and beverages consumed by respondents during online learning.

Types of Snacks/Beverages	Participants, n (%)
Eating foods or snacks	
No	304 (65.5)
Yes	160 (34.5)
Type of foods and snacks (n = 160)	
Prepared foods	16 (9.9)
Ready-to-eat savories	54 (33.59)
Bakery wares	33 (20.83)
Confectionery	31 (19.27)
Fruits, vegetables, seaweeds, nuts, and others	15 (9.38)
Drinking beverages	
No	86 (18.5)
Yes	378 (81.5)
Type of beverages (n = 378)	
Milk	63 (16.56)
Sugar-free tea or coffee	19 (4.91)
Tea or coffee with milk and sugar	115 (30.52)
Milk tea	34 (8.90)
Cocoa	32 (8.59)
Soft drinks	46 (12.12)
Juices	45 (11.81)
Others	25 (6.60)

Notes. n = numbers of participant.

**Table 5:** Multinomial logistic regression analysis of frequency of sugary beverage consumption and skipping breakfast associated with the duration of online learning (h/day) factor (n = 464).

Variables	Frequency of Sugary Beverage Consumption (>4 days/week) ^a^	Skipping Breakfast (≥3 times/week) ^b^
	OR (95%CI)	OR (95%CI)
Duration of online learning		
<3 h/day	1	1
3–6 h/day	0.42 * (0.235–0.758)	0.57 (0.258–1.256)
>6 h/day	0.47 (0.215–1.014)	0.29 * (0.116–0.730)

Notes. ^a^ The reference group was frequency of sugary beverage consumption (≤4 days/week). **^b^** The reference group was skipping breakfast (<3 times/week or none). * *p*-value < 0.01.

**Table 6:** Multinomial logistic regression analysis of the duration of sleep and quality of life in mental health associated with the duration of computer, tablet, and smartphone usage for online learning (h/day) factor (n = 464).

Variables	Duration of Sleep (h/Night) ^a^		Quality of Life in Mental Health ^b^	
	<6	6–8	Poor	Good
	OR (95%CI)	OR (95%CI)	OR (95%CI)	OR (95%CI)
Duration of computer, tablet, and smartphone usage for online learning				
<6 h/day	1	1	1	1
6–9 h/day	3.52 * (1.221–10.144)	2.50 (0.927–6.751)	3.74 * (1.066–13.082)	0.67 (0.392–1.159)
>9 h/day	4.62 **(1.542–13.842)	2.89 * (1.024–8.167)	5.29 ** (1.544–18.142)	0.73 (0.427–1.257)

Notes. ^a^ The reference group was hours of sleep >8 h/night. **^b^** The reference group was fair quality of life in mental health. * *p*-value < 0.05; ** *p*-value < 0.01.

## Data Availability

Not applicable.

## Supplementary Materials

The following supporting information can be downloaded at: https://www.mdpi.com/article/10.3390/nu14040890/s1, Table S1. Distribution of the food consumption behaviors, lifestyles, and quality of life in mental health of participants by duration of online learning (h/day) factor (n = 464). Table S2. Distribution of the food consumption behaviors, lifestyles, and quality of life in mental health of participants by duration of computer, tablet, and smartphone usage for online learning (h/day) factor (n = 464).
