# Supplementary material for: The Association between Online Learning and Food Consumption and Lifestyle Behaviors and Quality of Life in Terms of Mental Health of Undergraduate Students during COVID-19 Restrictions
Source: Nutrients. 2022 Feb 20;14(4):890. doi: 10.3390/nu14040890 (PMC8876014; doi:10.3390/nu14040890)
Supplement: Supplementary file 1 [file nutrients-14-00890-s001.zip › nutrients-1575743-supplementary.pdf]

Supplementary Table S1. Distribution of the food consumption behaviors, lifestyles, and quality of life in mental health of participants by duration of online learning (h/day) factor (n =464).

Variables	Duration of online learning, h/day			p-value (Chi-square)
	< 3	3-6	>6	
	n (%)	n (%)	n (%)	
Frequency of fruit and vegetable consumption				
≤ 4 days/week	35 (66.0)	251 (71.7)	43 (74.1)	0.635
> 4 days/week	18 (34.0)	102 (28.9)	15 (25.9)	
Fresh vegetable consumption				
<4 servings/day	38 (71.7)	239 (67.7)	37 (63.8)	0.673
≥4 servings/day	15 (28.3)	114 (32.3)	21 (36.2)	
Fruit consumption				
<3 servings/day	36 (67.9)	208 (58.9)	31 (53.4)	0.290
≥3 servings/day	17 (32.1)	145 (41.1)	27 (46.6)	
Frequency of high-fat diet consumption				
≤ 4 days/week	36 (67.9)	275 (77.9)	47 (81.0)	0.205
> 4 days/week	17 (32.1)	78 (22.1)	11 (19.0)	
Frequency of snack consumption				
≤ 4 days/week	44 (83.0)	309 (87.5)	51 (86.5)	0.644
> 4 days/week	9 (17.0)	44 (12.5)	7 (12.1)	
Frequency of western diet consumption				
≤ 4 days/week	48 (90.6)	340 (96.3)	53 (91.4)	0.077
> 4 days/week	5 (9.4)	13 (3.7)	5 (8.6)	
Frequency of sugary beverage consumption				
≤ 4 days/week	27 (50.9)	251 (71.1)	40 (69.0)	0.013*
> 4 days/week	26 (49.1)	102 (28.9)	18 (31.0)	
Frequency of instant food consumption				
≤ 4 days/week	48 (90.6)	340 (96.3)	57 (98.3)	0.089
> 4 days/week	4 (9.4)	13 (3.7)	1 (1.7)	
Eating foods or snacks during online learning				
No	34 (64.2)	234 (66.3)	36 (62.1)	0.802
Yes	19 (35.8)	119 (33.7)	22 (37.9)	
Drinking beverages during online learning				
No	13 (24.5)	60 (17.0)	13 (22.4)	0.302
Yes	40 (75.7)	293 (83.0)	45 (77.6)	
Duration of exercise				
No exercise	32 (60.4)	187 (53.0)	30 (51.7)	0.476
< 3 times or 150 min/week	16 (30.2)	116 (32.9)	16 (27.6)	
≥ 3 times or 150 min/week	5 (9.4)	50 (14.2)	12 (20.7)	
Self-cooking				
No	37 (69.8)	210 (59.5)	34 (58.5)	0.340
Yes	16 (30.2)	143 (40.5)	24 (41.4)	
Skipping breakfast				
< 3 times/week or none	28 (15.1)	84 (23.8)	22 (37.9)	0.016*
≥ 3 times/week	45 (84.9)	269 (76.2)	36 (62.1)	
The quality of life in mental health				
Poor	9 (17.0)	31 (8.8)	8 (13.8)	0.301
Fair	30 (56.6)	231 (65.4)	38 (65.5)	
Good	14 (26.4)	91 (25.8)	12 (20.7)	

Notes. n = numbers of participant; **p*-value < 0.05

Supplementary Table S2. Distribution of the food consumption behaviors, lifestyles, and quality of life in mental health of participants by duration of computer, tablet, and smartphone usage for online learning (h/day) factor (n =464).

Variables	Duration of computer, tablet, and smartphone usage for online learning, h/day			p-value (Chi-square)
	< 6	6-9	>9	
	n (%)	n (%)	n (%)	
Frequency of fruit and vegetable consumption				
≤ 4 days/week	80 (74.8)	117 (66.5)	132 (72.9)	
> 4 days/week	27 (25.2)	59 (33.5)	49 (27.1)	0.246
Fresh vegetable consumption				
< 4 servings/day	75 (70.1)	124 (70.5)	115 (63.5)	
≥ 4 servings/day	32 (29.9)	52 (29.5)	66 (36.5)	0.313
Fruit consumption				
< 3 servings/day	61 (57.0)	107 (60.8)	107 (59.1)	
≥ 3 servings/day	46 (43.0)	69 (39.2)	74 (40.9)	0.820
Frequency of high-fat diet consumption				
≤ 4 days/week	83 (77.6)	142 (80.7)	133 (73.5)	
> 4 days/week	24 (22.4)	34 (19.3)	48 (26.5)	0.267
Frequency of snack consumption				
≤ 4 days/week	94 (87.9)	155 (88.1)	155 (85.6)	
> 4 days/week	13 (12.1)	21 (11.9)	26 (14.4)	0.762
Frequency of western diet consumption				
≤ 4 days/week	105 (98.1)	170 (96.6)	166 (91.7)	
> 4 days/week	2 (1.9)	6 (3.4)	15 (8.3)	0.052
Frequency of sugary beverage consumption				
≤ 4 days/week	72 (67.3)	130 (73.9)	116 (64.1)	
> 4 days/week	35 (32.7)	46 (26.1)	65 (35.9)	0.132
Frequency of instant food consumption				
≤ 4 days/week	103 (96.3)	171 (97.2)	171 (94.5)	
> 4 days/week	4 (3.7)	5 (2.8)	10 (5.5)	0.431
Eating foods or snacks during online learning				
No	79 (73.8)	116 (65.9)	109 (60.2)	
Yes	28 (26.2)	60 (34.1)	72 (39.8)	0.063
Drinking beverages during online learning				
No	22 (20.6)	36 (20.5)	28 (15.5)	
Yes	85 (79.4)	140 (79.5)	153 (84.5)	0.397
Duration of sleep				
< 6 h/night	25 (23.4)	56 (31.8)	63 (34.8)	
6-8 h/night	71 (66.4)	113 (64.2)	112 (61.9)	0.037*
> 8 h/night	11 (10.3)	7 (4.0)	6 (3.3)	
Duration of exercise				
No exercise	59 (55.1)	84 (47.7)	106 (58.6)	
< 3 times or 150 min/week	29 (27.1)	67 (38.1)	52 (28.7)	0.156
≥ 3 times or 150 min/week	19 (17.7)	25 (14.2)	23 (12.7)	
Self-cooking				
No	72 (67.3)	98 (55.7)	111 (61.3)	
Yes	35 (32.7)	78 (44.3)	70 (38.7)	0.148
Skipping breakfast				
< 3 times/week or none	26 (24.3)	50 (28.4)	38 (21.0)	
≥ 3 times/week	81 (75.7)	126 (71.6)	143 (79.0)	0.265
The quality of life in mental health				
Poor	3 (2.8)	19 (10.8)	26 (14.4)	
Fair	69 (64.5)	117 (66.5)	113 (62.4)	0.017*
Good	35 (32.7)	40 (22.7)	42 (23.2)	

Notes. n = numbers of participant; *p-value < 0.05
